# Lifestyle Characteristics and Gene Expression Analysis of *Colletotrichum camelliae* Isolated from Tea Plant [*Camellia sinensis* (L.) O. Kuntze] Based on Transcriptome

**DOI:** 10.3390/biom10050782

**Published:** 2020-05-18

**Authors:** Fei Xiong, Yuchun Wang, Qinhua Lu, Xinyuan Hao, Wanping Fang, Yajun Yang, Xujun Zhu, Xinchao Wang

**Affiliations:** 1 College of Horticulture, Nanjing Agricultural University, No.1 Weigang, Nanjing 210095, China; 2017104088@njau.edu.cn (F.X.); fangwp@njau.edu.cn (W.F.); 2 Tea Research Institute, Chinese Academy of Agricultural Sciences; National Center for Tea Improvement; Key Laboratory of Tea Biology and Resources Utilization, Ministry of Agriculture and Rural Affairs of the People’s Republic of China, Hangzhou, 310008, China; ycwang0201@126.com (Y.W.); lqhazbc@163.com (Q.L.); haoxy@tricaas.com (X.H.); yjyang@tricaas.com (Y.Y.); 3 College of Agriculture and Food Sciences, Zhejiang A&F University, Lin’an, Hangzhou 311300, China

**Keywords:** *Colletotrichum camelliae*, tea plant, infection mechanism, transcriptome

## Abstract

*Colletotrichum camelliae* is one of the most serious pathogens causing anthracnose in tea plants, but the interactive relationship between *C. camelliae* and tea plants has not been fully elucidated. This study investigated the gene expression changes in five different growth stages of *C. camelliae* based on transcriptome analysis to explain the lifestyle characteristics during the infection. On the basis of gene ontology (GO) enrichment analyses of differentially expressed genes (DEGs) in comparisons of germ tube (GT)/conidium (Con), appressoria (App)/Con, and cellophane infectious hyphae (CIH)/Con groups, the cellular process in the biological process category and intracellular, intracellular part, cell, and cell part in the cellular component category were significantly enriched. Hydrolase activity, catalytic activity, and molecular_function in the molecular function category were particularly enriched in the infection leaves (IL)/Con group. Kyoto Encyclopedia of Genes and Genomes (KEGG) analysis indicated that the DEGs were enriched in the genetic information processing pathway (ribosome) at the GT stage and the metabolism pathway (metabolic pathways and biosynthesis of secondary metabolism) in the rest of the stages. Interestingly, the genes associated with melanin biosynthesis and carbohydrate-active enzymes (CAZys), which are vital for penetration and cell wall degradation, were significantly upregulated at the App, CIH and IL stages. Subcellular localization results further showed that the selected non-annotated secreted proteins based on transcriptome data were majorly located in the cytoplasm and nucleus, predicted as new candidate effectors. The results of this study may establish a foundation and provide innovative ideas for subsequent research on *C. camelliae*.

## 1. Introduction

Tea is one of the most popular non-alcoholic drinks in the world. China is the largest producer and consumer of tea in the world [1]. Tea leaves, which are the raw material of tea products, are usually infected by several pathogens. Tea anthracnose, primarily caused by *Colletotrichum camelliae*, is one of the serious diseases in tea plant [*Camellia sinensis* (L.) O. Kuntze] [2]. However, the process and infection mechanism of *C. camelliae* in tea plant has not been thoroughly characterized.

*Colletotrichum* spp. has long served as a model system for the hemi-biotrophic pathogen that causes anthracnose in woody and herbaceous plants [3,4], which cause different pathological phenotypes via a multistage infection process. *Colletotrichum* spp. has two types of infection, one of which is represented by *C. higginsianum*, which directly penetrates the host cells and immediately produces secondary hyphae from the primary hyphae in the biotrophic stage. In comparison, another one is represented by *C. graminicola*, whereby the primary hyphae colonize as many host cells as possible after the spikes enter the host plants. Secondary hyphae form from the primary hyphae only when growth conditions settle down. Secondary hyphae formation starts to kill the cells and turns into the necrotrophic stage [5].

1,8-Dihydroxynaphthalene (1,8-DHN) melanin is the main source of melanin in most fungal pathogens, including *Colletotrichum* spp [6]. Appressoria play an important role in pathogenicity because they provide a solid base for the penetration hyphae to puncture the cuticle and cell walls [7], which require melanization of appressoria. This melanization leads to the selective penetration of the cell wall, resulting in a huge swelling pressure, which provides the impetus for the physical defense of the hyphae to penetrate the plant epidermis. This process also needs cyclic adenosine monophosphate (cAMP) signalling and the mitogen-activated protein kinase (MAPK) signalling pathway [8,9,10,11]. Appressoria can secrete various virulence proteins into the host tissue [12], such as carbohydrate-active enzymes, serine proteases, and cytochrome P450 enzymes [5,13,14,15]. Carbohydrate-active enzymes (CAZy), including plant cell wall-degrading enzymes (PCWDEs), function in building and breaking down complex carbohydrate and glycoconjugates. In addition, CAZys are associated with plant cell wall degradation, which can boost the infectious capacity of pathogenic fungi [16].

Because of the massive yield loss caused by *Colletotrichum* spp. to agriculture, chemical and biological pesticides are frequently used in crops, including tea plants [17,18]. Therefore, clarifying the process of *Colletotrichum* spp. infection is vital for the prevention and treatment of anthracnose in tea plants. To defend against pathogen infection, plants evolve a highly complex immune system. Pathogens are mainly perceived by pattern-triggered immunity (PTI) and effector-triggered immunity (ETI) systems [19,20]. In contrast, the pathogen can generally inhibit PTI by secreting virulence factors (effectors) or ETI [21]. The wide-ranging polymorphic family of intracellular nucleotide-binding/leucine-rich repeats (NLRs) specifically recognizes effectors and triggers the downstream defense response again [20,21,22]. In this effect, pathogens evolve different types of effectors to manipulate the physiology of host plants to avoid capture by the plant immune system. Therefore, research on the effector function of pathogens is critical to understanding their pathogenicity, including the functional location of effectors. However, the identification of key effectors of *C. camelliae* in tea plants warrants further research.

*C. camelliae* is currently known as a dominant fungal pathogen that occurs on *Camellia* plants [23]. In the early stage of infection, there will be tiny water stain symptoms on the tea leaves. With the increase of the infection time, the water-soaked lesions gradually expand and become necrotic. In this study, we utilized transcriptome data of five infection-related stages (resting conidia, germ tube, appressoria, cellophane infection hyphae, and infected leaves) of *C. camelliae* to reveal the variation of the gene expression in the process of the infection and preliminarily classified the candidate effectors. The primary research concerning *C. camelliae* will first provide information from the pathogen side. Moreover, this study may provide a foundation for future research investigating the host specificity of *C. camelliae* to tea plants, as well as other crops.

## 2. Materials and Methods

### 2.1. Sample Preparation

Conidial, conidiophore, and appressorium characteristics were determined using methods described by Liang et al. [24]. The *C. camelliae* strain LS-19 were used for this research [2,25]. **Conidium (Con)**: The strain was inoculated into a PDA plate (potato dextrose agar) and grown for 4 days. The edge of the colony was punched with a puncher, and mycelial discs were placed into PDB (potato dextrose broth) and shaken at 28 °C and 180 rpm to breed spores. After sporulation, the broth was filtered twice, using a Miracloth membrane to remove mycelia, and centrifuged at 10,000 rpm for 5 min; the supernatant was discarded, and the pellet was washed twice with sterile water. The pellet was resuspended in sterile water and then centrifuged at 10,000 rpm for 5 min, and the supernatant was discarded to obtain spores. Purified conidia were immediately frozen in liquid nitrogen and stored at −80 °C. **Germ tube (GT)**: The conidia were placed in a 50-mL centrifuge tube and resuspended with 30 mL PDB. The conidia concentration was adjusted to 1 × 10^7^/mL, and the mixture was shaken at 25 °C and 180 rpm for 2 hpi (hours post-inoculation). After centrifugation at 10,000 rpm for 5 min, the supernatant was discarded, and the germ tubes were washed twice with sterile water. Purified germ tubes were immediately frozen in liquid nitrogen and stored at −80 °C. **Appressoria (App)**: Appressoria were induced by conidia suspension (1 × 10^7^/mL) on a 1 cm^2^ diameter layer of cellophane, whereby 200 μL of conidial suspension was dropped onto cellophane and covered on PDA medium, then incubated at 25 °C, 80% humidity in darkness for 8 hpi. Appressoria colonized on the cellophane, and the cellophane was removed from the PDA medium and immediately frozen in liquid nitrogen and stored at −80°C. **Cellophane infectious hyphae (CIH)**: The approach for obtaining CIH was the same as that for appressoria with different treatment times. At 36 hpi, the cellophanes were removed and immediately frozen in liquid nitrogen and stored at −80 °C. **Infection Leaf (IL)**: The tea branches (*C. sinensis* cv. *Longjing43*, LJ43) were harvested, surface-rinsed with tap water, and soaked with 75% ethanol. Finally, the tea branches were rinsed with sterile water. The branches were placed in erlenmeyer flasks with sterile water and immediately used for inoculation, for which the conidial suspension (1 × 10^7^/mL in 0.1% Tween 20) was sprayed onto the leaves which were then incubated in a moist chamber at 25 °C with 80% humidity. After 48 hpi, black waterstain-like lesions gradually appeared around the wounded area. At 48 hpi, ten leaf lesions were cut with scissors then harvested without healthy tissue. The samples were frozen in liquid nitrogen and then stored at −80 °C for RNA extraction. Three biological replicates were collected for five samples.

### 2.2. Microscopy Observation

Microscopic examinations (Eclipse 80i, Nikon, Tokyo, Japan) were conducted to determine the growth stages of *C*. *camelliae* before sampling. 20 µL of the conidia suspension in 2.1 were dropped onto the slide for microscope observation. GT tissue samples were examined in the same way as the Con. The cellophane incubating App was removed from the PDA medium and placed on a slide to observe whether a large number of appressoria structures were produced. CIH samples were examined in the same way as the App.

### 2.3. Pathogen Infection Assay

Tea plant of the cultivar Longjing 43 (LJ43) and the *C. camelliae* LS-19 strain were used for the assay [25]. The isolated second leaves at the tip of the branches of three-year-old tea plants were put under needle wound treatment [26], then inoculated with conidial suspensions of *C. camelliae* (10^7^ spores/mL), and the petioles were covered with wet cotton. The leaves were then maintained in the glasshouse at 25 ± 0.5 °C and 80% humidity. Three individual leaves were placed in a Petri dish as a group, and one group means one biological duplication. The leaves were harvested at 12, 24, 48, and 72 hpi and frozen in liquid nitrogen immediately. The samples were used for the RNA extraction and qRT-PCR test.

### 2.4. RNA Isolation and Sequencing

Total RNA was extracted using the CTAB (hexadecyltrimethylammonium bromide) method as previously described [27]. RNA degradation and contamination were monitored with 1% agarose gel analysis. RNA concentration was measured using a Qubit^®^ RNA Assay Kit in a Qubit^®^ 2.0 Fluorometer (Life Technologies, Waltham, CA, USA), and RNA integrity was assessed using the RNA Nano 6000 Assay Kit of the Agilent Bioanalyzer 2100 system (Agilent Technologies, California, CA, USA) [24].

### 2.5. Transcriptome Data Analysis

A total of 3.0 μg RNA per sample was used as input material for RNA sequencing. Library preparation and library sequencing were performed at the Novogene Bioinformatics Institute (Beijing, China) on the Illumina HiSeqTM 2000 platform (Illumina, San Diego, CA, USA), and paired-end reads measuring 150 bp in length were yielded. Based on the requirements of the library, the low-quality reads were removed. The index of the reference genome (*C. camelliae* LS-19, unpublished) was built using Hisat2 v2.0.5 [28], and paired-end clean reads were aligned to the reference genome using Hisat2 v2.0.5. Gene expression quantification was performed using featureCounts v1.5.0-p3 to count the read numbers mapped to each gene [29]. Then, the FPKM (Fragments Per Kilobase of transcript sequence per Millions base pairs sequenced) of each gene was calculated based on the length of the gene and read count mapped to this gene. FPKM, the expected number of fragments per kilobase of transcript sequence per million base pairs sequenced, is currently the most commonly used method for estimating gene expression levels [30].

Differentially expressed gene (DEG) analysis of two conditions/groups (three biological replicates per condition) was performed using the DESeq2 R package (1.16.1) [31]. The resulting *p*-values were adjusted using Benjamini and Hochberg’s approach for controlling the false discovery rate. Genes with an adjusted *p*-value < 0.01 and log_2_FoldChange > 2 were calculated using DESeq2 and considered to be differentially expressed. The clusterProfiler R package [32] was used to test the statistical enrichment of DEGs (*p*-value < 0.05, log_2_FoldChange > 2) in the Kyoto Encyclopedia of Genes and Genomes (KEGG) pathways (https://www.genome.jp/kegg/) with KEGG enrichment analysis using Pathway in the KEGG database as the unit. A hypergeometric test was used to identify pathways with significant enrichment in DEGs compared with the genome background. Gene ontology (GO) enrichment analysis of DEGs (*p*-value < 0.05, log_2_FoldChange > 2) was implemented using the clusterProfiler R package with the GOseq method [33].

### 2.6. qRT-PCR Verification Assay for Candidate Effector Proteins (CEPs)

To verify the expression of CEPs, pathogen infection samples were used for qRT-PCR assays. One microgram of total RNA was used for cDNA synthesis. A PrimeScript RT enzyme with a gDNA eraser (Takara, Osaka, Japan) was used for cDNA synthesis. qRT-PCR was performed on an Applied Biosystems 7500 Sequence Detection System using SYBR Premix Ex TaqTM II (Takara, Kyoto, Japan). The primers were designed on NCBI and are listed in Table S1. The internal control gene *Ccnew1* was described by He et al. [34], the biomass correction of fungal and plant were referred to by He et al. and Vieira et al. [34,35], and the relative expression levels were calculated using the 2^−ΔΔCt^ method [36].

### 2.7. Cloning and Subcellular Localization of Candidate Effectors

The original sequence information was from the *C. camelliae* genome (unpublished data from our lab). Primer 5 software (Primier, San Francisco, CA, USA) was used to design the primers (Table S2). The KOD-NEO-PLUS cloning kit (TOYOBO, Osaka, Japan) was used for gene cloning. The procedures followed the kit instructions. TMHMM (http://www.cbs.dtu.dk/services/TMHMM/) was used to analyze the transmembrane sequences of candidate effector proteins, and the primers were designed to amplify the signal peptide region and add a starting codon ATG at the 5’ terminal. pBin-eGFP vector was used for subcellular localization, and seamless cloning method was used to connect the target fragment with the linear vector, following the instructions of the GBclonart Seamless Cloning Kit (Genebank Biosciences Inc., Suzhou, China). The seamless cloning primers are shown in Table S3. The successfully constructed subcellular localization vector was introduced into *agrobacterium tumefaciens* GV3101 using the liquid nitrogen quick-freezing method [37]. pBin-eGFP was unloaded into *agrobacterium tumefaciens* as a negative control. The activated *agrobacterium tumefaciens* was cultured to OD_600_ = 1, centrifuged at 3000 rpm for 5 min, then re-suspended with infection solution (Table S4) to OD_600_ = 1. The pH was adjusted to 5.7 with KOH/HCl [38]. These constructs were transfected into 5-week-old *Nicotiana benthamiana* leaves (with nuclear marker) after being placed at 28 °C for 3 h. The GFP fluorescence signal was observed with an FV1000 confocal microscope (Olympus, Tokyo, Japan). Excitation wavelengths of nuclear markers RFP and eGFP were 532 nm and 488 nm, respectively.

## 3. Results

### 3.1. Different Developmental Stages of C. camelliae

**Figure 1:**
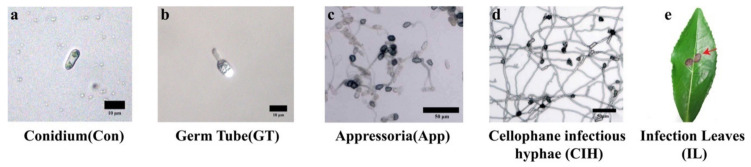
Morphology detection of *C. camelliae* in the different growth stages. (**a**) conidium (Con) morphology, bars = 10 μm; (**b**) germ tube (GT) morphology (2 hpi), bars = 10 μm; (**c**) dark-domed appressorium (App) morphology (8 hpi), bars = 50 μm; (**d**) cellophane infectious hyphae (CIH) morphology (36 hpi), bars = 50 μm; (**e**) infection leaf (IL) lesion morphology (72 hpi). (hpi: hours post-inoculation).

According to the microscopy detection, different growth stages of *C. camelliae* were observed in vitro (Figure 1). Conidium (Con) morphology was hyaline, smooth, and septate (Figure 1a). Liquid PDB culture for inducing conidial gemmation, PDB culture with 10^7^ per mL conidial concentration at collection time (2 hpi), and microscopic examination revealed about 93.3% of conidia produce germ tubes (Figure 1b). The diameter layer induced appressoria (App), the cellophane was transferred to the slide, and dark dome-shaped appressoria was observed (Figure 1c). About 71.8% of the conidia produced appressorium structure and cellophane infectious hyphae (CIH) at 36 hpi. The CIH protrude from appressoria or primary hyphae and spread randomly (Figure 1d). The infection leaves (IL) were incubated with conidia (10^7^ per mL) and collected at 72 hpi, and the dark brown spots (Figure 1e) were small water stains and gradually expanded with time.

### 3.2. Transcriptome Assembly and Identification of Differentially Expressed Genes (DEGs) among Different Growth Stages

**Figure 2:**
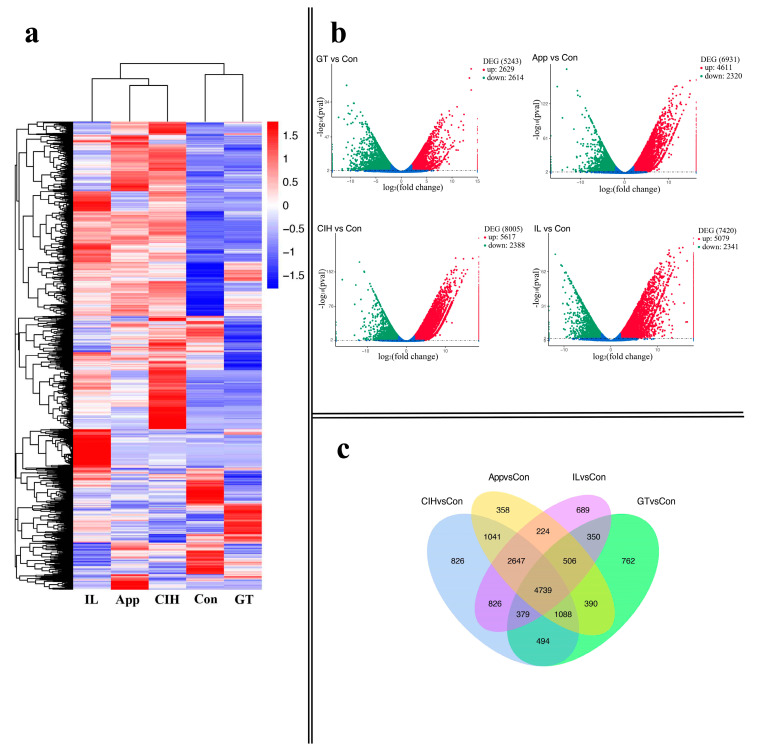
Identification and expression profile analyses of differentially expressed genes (DEGs). (**a**) Expression profile and clustering of DEGs in different growth stages of *C. camelliae*. (**b**) The number of up-regulated and down-regulated DEGs was compared with Con at different growth stages. (**c**) Overlapping relationship of DEGs between different comparable groups. (Con: conidium; GT: germ tube; App: appressoria; CIH: cellophane infectious hyphae; IL: infection leaves).

A total of 940,900,502 clean RNA-seq reads (NCBI accession number: PRJNA628751) were obtained and aligned to the reference genome of *C. camelliae*. The intended sequencing depth for App was 7 Gb clean bases per library; GT was 9 Gb clean bases per library; Con and CIH were 10 Gb clean bases per library, and the IL was 11 Gb per library. The quality of the data output is shown in Table S5. The Pearson correlation analysis showed the Pearson correlation coefficient was >0.92 between biological repeats (Figure S1). There was hierarchical clustering of five clusters of DEGs according to the log_10_^(FPKM+1)^ values. This analysis identified five group of genes that were specifically strongly upregulated compared to the other four groups, indicating that *C. camelliae* has a high number of DEGs in different growth stages, suggesting different biological activities at these phases. (Figure 2a). The DEGs were analysed using three independent biological repeats for each group under *p*-value < 0.01, log_2_FoldChange > 2. A total of 5243, 6931, 8005, and 7420 DEGs were detected between GT and Con, App and Con, CIH and Con, and IL and Con, respectively; the number of upregulated and downregulated genes was 2629, 4611, 5617, 5079 and 2614, 2320, 2388, 2341, respectively; the volcano plot showed that the number of upregulated genes in CIH was higher than that in GT, App, and IL; in contrast, the number of upregulated genes in GT was the lowest (Figure 2b). We particularly compared App and CIH, which are both vital stages for invasion, and we found that 1047 DEGs were upregulated and 2391 DEGs were downregulated when App was used as a mock control (Figure S2). The Venn diagram analysis of DEGs showed overlapping relationships of four comparison groups, which indicated 4739 DEGs were shared among them (Figure 2c).

### 3.3. Functional Enrichment Reveals the Importance of Cellular Processes and Metabolic Pathways

**Figure 3:**
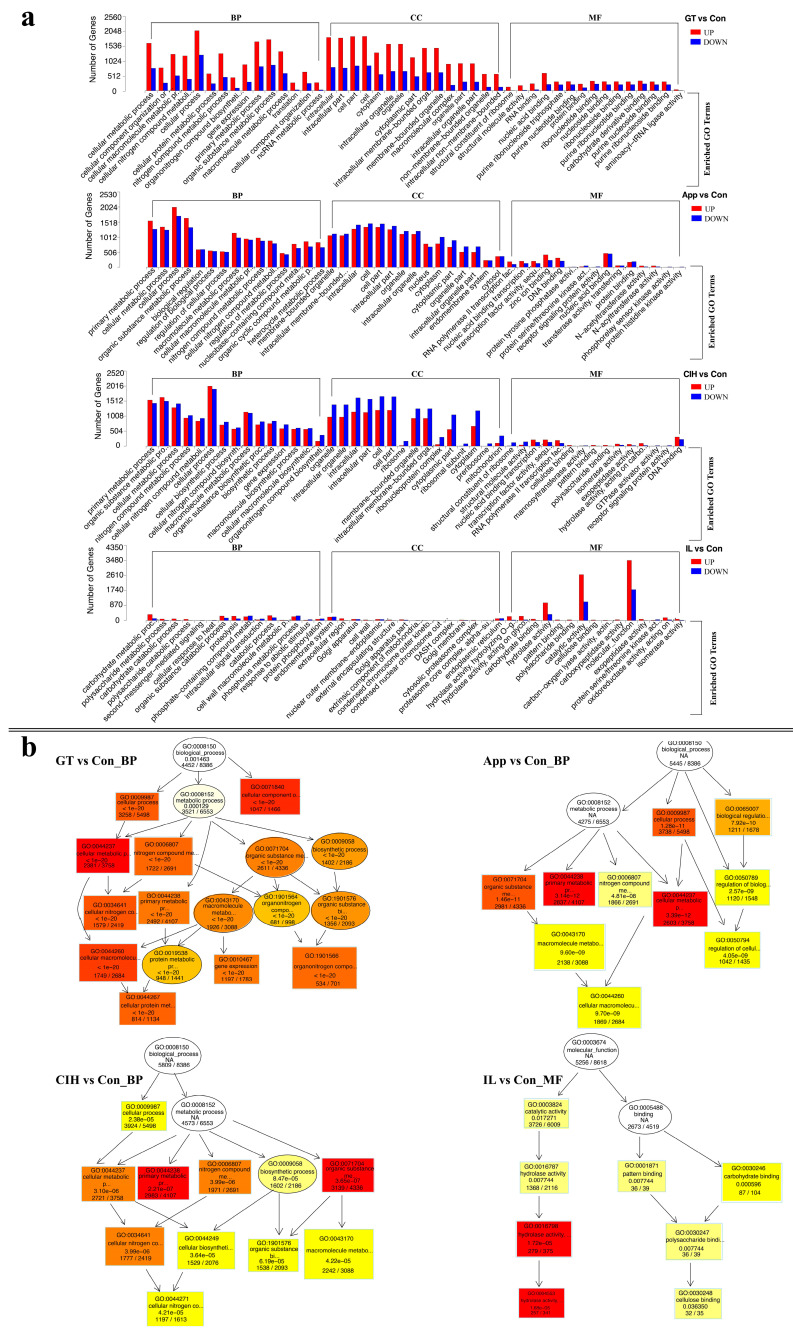
Gene ontology (GO) analysis of all differentially expressed genes (DEGs) in different growth stages. (**a**) GO enrichment of four comparable groups in different growth stages of *C. camelliae* revealed functional classification of all DEGs; red column means the number of up-regulated DEGs, blue column means the down-regulated DEGs. (**b**) TopGO analysis of key GO terms in different growth stages of *C. camelliae*; the color represents the degree of enrichment: the brighter the color, the higher the degree of enrichment. (Con: conidium; GT: germ tube; App: appressoria; CIH: cellophane infectious hyphae; IL: infection leaves; BP: biological process; CC: cellular component; MF: molecular function).

To assess the biological activities of *C. camelliae* in the five different life-growth stages, all DEGs were used for GO and KEGG enrichment, and up-DEG-based GO and KEGG analyses were performed as supplementary information (Figure S3). In each category, we selected the top 15 GO terms enriched by the DEGs in comparisons of GT/Con, App/Con, CIH/Con, and IL/Con (Figure 3a and Table S6). In the biological process category, the cellular process (GO:0009987) term was highly enriched in GT/Con, App/Con, and CIH/Con groups, and in the cellular component category, the intracellular (GO:0005622), intracellular part (GO:0044424), cell (GO:0005623), and cell part (GO:0044464) terms were highly enriched in in GT/Con, App/Con, and CIH/Con groups. Interestingly, in the GT/Con group, the number of up-DEGs in each term is greater than the number of down-DEGs. In the molecular function category, the hydrolase activity (GO:0016787), catalytic activity (GO:0003824), and molecular_function (GO:0003674) terms were highly enriched in the IL/Con group. Overall, the GT/Con, App/Con, and CIH/Con groups were mainly enriched in the biological process and cellular component categories, but the IL/Con group was mainly enriched in molecular function category. The subsequent topGO (graphical display of GO enrichment analysis) analysis was performed through the key component of the biological process in GT/Con, App/Con, and CIH/Con followed by the molecular function for IL/Con (Figure 3b). The results of GT/Con, App/Con, and CIH/Con showed that the DEGs involved in the “primary metabolic process” (GO:0044238) were 60.7%, 69.1%, and 72.6%, respectively. In contrast, the IL/Con results showed that the number of DEGs involved in “hydrolase activity, hydrolysing O-glycosyl compounds” (GO:0004553) was 75.4%.

**Figure 4:**
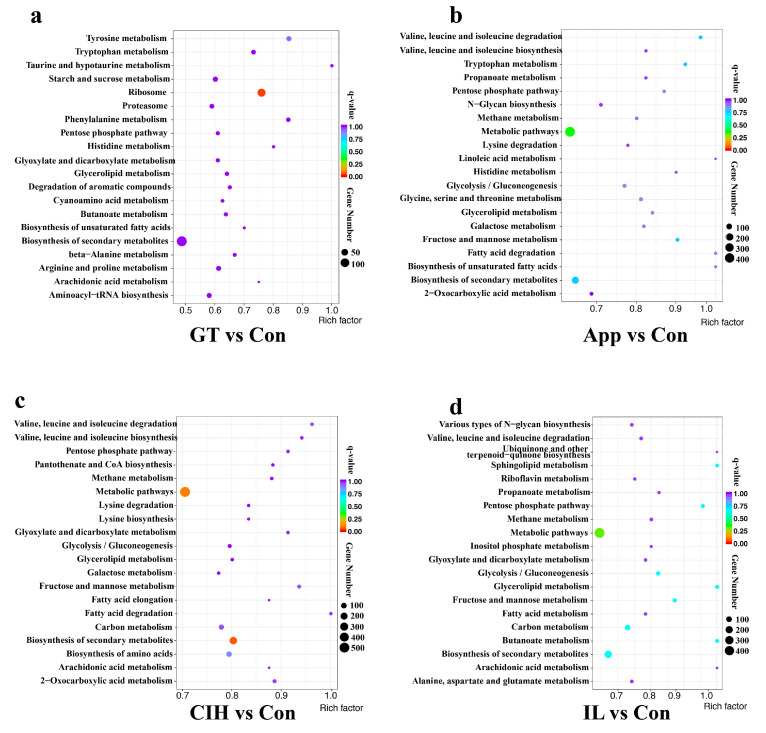
Kyoto Encyclopedia of Genes and Genomes (KEGG) analysis of all differentially expressed genes (DEGs) in four comparable groups of *C. camelliae*. (**a**) KEGG analysis of DEGs in GT/Con group; (**b**) KEGG analysis of DEGs in App/Con group; (**c**) KEGG analysis of DEGs in CIH/Con group; (**d**) KEGG analysis of DEGs in IL/Con group. (Con: conidium; GT: germ tube; App: appressoria; CIH: cellophane infectious hyphae; IL: infection leaves).

The KEGG cluster analysis of all DEGs is shown in Figure 4. The data show that the number of genes annotated by the biosynthesis of secondary metabolism was the largest in the GT/Con group. The number of genes annotated by the metabolic pathway was the largest in the three remaining comparison groups. Notably, the number of genes involved in the biosynthesis of secondary metabolism was also significant in the CIH/Con group. We subsequently analysed the KEGG enrichment of upregulated DEGs of these four comparison groups (Figure S3a). Interestingly, the ribosome became the most DEG enriched pathway in the GT/Con group. In the App/Con group, the starch and sucrose metabolism pathway enriched dozens of genes with high rich factors. Most of the genes enriched in the CIH and IL comparison groups are related to metabolic pathway and biosynthesis of secondary metabolism. The genes relevant to the metabolic pathway and biosynthesis of secondary metabolism function in the penetration and development of secondary hyphae, such as melanization-dependent genes, cytochrome P450 enzyme genes, fatty acid coding genes [5,39], and cytotoxic activity genes, and these metabolic processes are vital for the infection and necrotrophic stage. The specific information of genes enriched by different KEGG pathways is shown in Table S7.

### 3.4. Infection Strategy during Different Infection Stages

**Figure 5:**
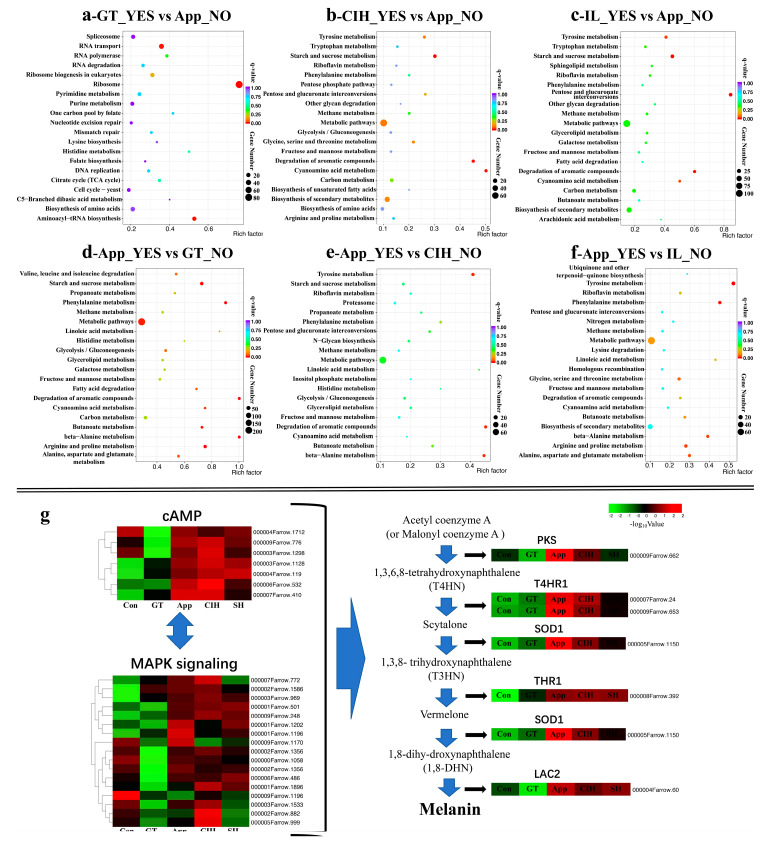
Kyoto Encyclopedia of Genes and Genomes (KEGG) analysis of up-DEGs (differentially expressed genes) in the GT/App, CIH/App and IL/App groups and expression patterns of melanin biosynthesis related genes. (**a**–**f**). KEGG analysis of specific up-regulated DEGs in different comparable groups of *C. camelliae*. (**g**) Heat map of relevant melanin biosynthesis genes of *C. camelliae*; the expression was based on the FPKM (Fragments Per Kilobase of transcript sequence per Millions base pairs sequenced) value. Color scale was provided for two figures. (cAMP: cyclic adenosine monophosphate; MAPK: mitogen-activated protein kinase; Con: conidium; GT: germ tube; App: appressoria; CIH: cellophane infectious hyphae; IL: infection leaves; *PKS*: *Polyketide synthase*; *T4HR1*: *1,3,6,8-tetrahydroxynaphthalene reductase 1*; *SOD1*: *Scytalone dehydratase 1*; *THR1*: *1,3,8-trihydroxynaphthalene reductase 1*; *LAC2*: *Laccase gene 2*).

In this study, we divided the growth stage of *C. camelliae* into five stages. We identified the DEGs by comparison with the Con stage, produced a Venn diagram showing the specific up-DEGs in the GT/App, CIH/App, and IL/App groups (Figure S4), and then utilize specific up-regulated genes in different comparison groups to performed KEGG analysis (Table S8). In the GT stage, there were 1023 upregulated DEGs compared to the App stage. KEGG analysis enriched 657 genes in several biochemical pathways. Most metabolic pathways were enriched in genetic information processing (e.g., ribosome, aminoacyl-tRNA biosynthesis, RNA transport, and DNA replication), indicating that the reproduction of *Colletotrichum* starts at the GT stage (Figure 5a). For the App stage, most of the upregulated genes were enriched in metabolic pathways, such as carbohydrate metabolism (e.g., starch and sucrose metabolism, glycolysis/gluconeogenesis) and lipid metabolism (e.g., fatty acid degradation) (Figure 5b). In contrast, the upregulated genes in CIH and IL were enriched in the metabolic pathway and biosynthesis of secondary metabolism (Figure 5c,e). Specific upregulated genes in the App stage were not enriched in the biosynthesis of the secondary metabolites pathway (Figure 5d). Compared to the IL, the upregulated DEGs in the App stage were mainly focused on amino acid metabolism (e.g., tyrosine metabolism, lysine degradation, glycine, serine, and threonine metabolism) (Figure 5f). In the two comparison groups (Figure 5e,f), we observed that the tyrosine metabolism pathway was enriched but absent in the App_YES vs. GT_NO group.

We identified several key genes (e.g., *PKS*, *THR1*, *T4HR1*, *SCD1*, and *LAC2*), using gene annotation, that function in the process of melanin biosynthesis (Table S9) [6,40,41]. These key genes were upregulated in the App stage and maintain a high level of expression in the CIH and IL stages. Putative transcripts encoding cAMP-dependent kinases and MAPK signalling pathways were identified (Table S10). Most of the genes relevant to cAMP and MAPK signalling were activated for the preparation of melanin biosynthesis. Interestingly, we noticed that several cAMP and MAPK-related genes were suppressed in the GT stage but with higher expression in the Con stage, indicating the unusual biological process between the Con and App phases in *C. camelliae* (Figure 5g).

Polysaccharides and carbohydrates are the main components of the plant cell wall, and the pathogen of CAZys is the main tool for decomposition of plant cell walls [16]. Among 278 DEGs belonging to carbohydrate-active enzyme classes, 288 carbohydrate-active enzyme-associated modules were predicted in this study (Figure S5 and Table S11) [42]. CAZys are mainly divided into 5 classes: glycoside hydrolases (GHs), glycosyl transferases (GTs), polysaccharide lyases (PLs), carbohydrate esterases (CEs), and auxiliary activities (AAs). These enzymes include carbohydrate-binding modules (CBMs). FPKM-based expression clustering showed that GHs, GTs, CEs, AAs, and CBMs were upregulated or partly upregulated at the App and CIH stages. Interestingly, PLs were upregulated only in the IL stage. Moreover, a small group of CAZys showed high expression at the Con stage.

### 3.5. Prediction of Candidate Effector Proteins (CEPs) and Subcellular Localization Assay

**Figure 6:**
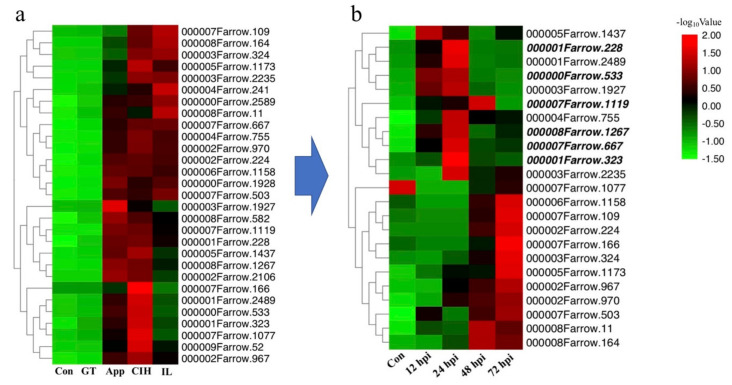
Expression analysis of candidate effector proteins (CEPs). (**a**) Heat map of CEPs of *C. camelliae* based on the FPKM (Fragments Per Kilobase of transcript sequence per Millions base pairs sequenced). (**b**) Heat map of CEPs based on qRT-PCR assay in vivo. Color scale was provided for two figures. (Con: conidiun; GT: germ tube; app: appressoria; CIH: cellophane infectious hyphae; IL: infection leaves; hpi: hours post-inoculation).

Based on the transcriptome data, we predicted 29 secreted proteins as potential candidate effectors that were significantly upregulated continuously based on the FPKM value (Figure 6a and Table S12). We subsequently performed qRT-PCR assays. Except for 6 genes (i.e., *Cc52*, *Cc241*, *Cc582*, *Cc1928*, *Cc2106*, and *Cc2589*), the expression of all other genes was detected. The qRT-PCR data indicated that several genes showed high expression at 24 h and 48 h after infection (Figure 6b). Then, we cloned six candidate effectors (i.e., *Cc228*, *Cc323*, *Cc533*, *Cc667*, *Cc1119*, and *Cc1267*) (Table S13) whose function remains unknown and may be closely related to this host specialization. Signal prediction showed that they all have signal peptides at the front of the C terminus.

**Figure 7:**
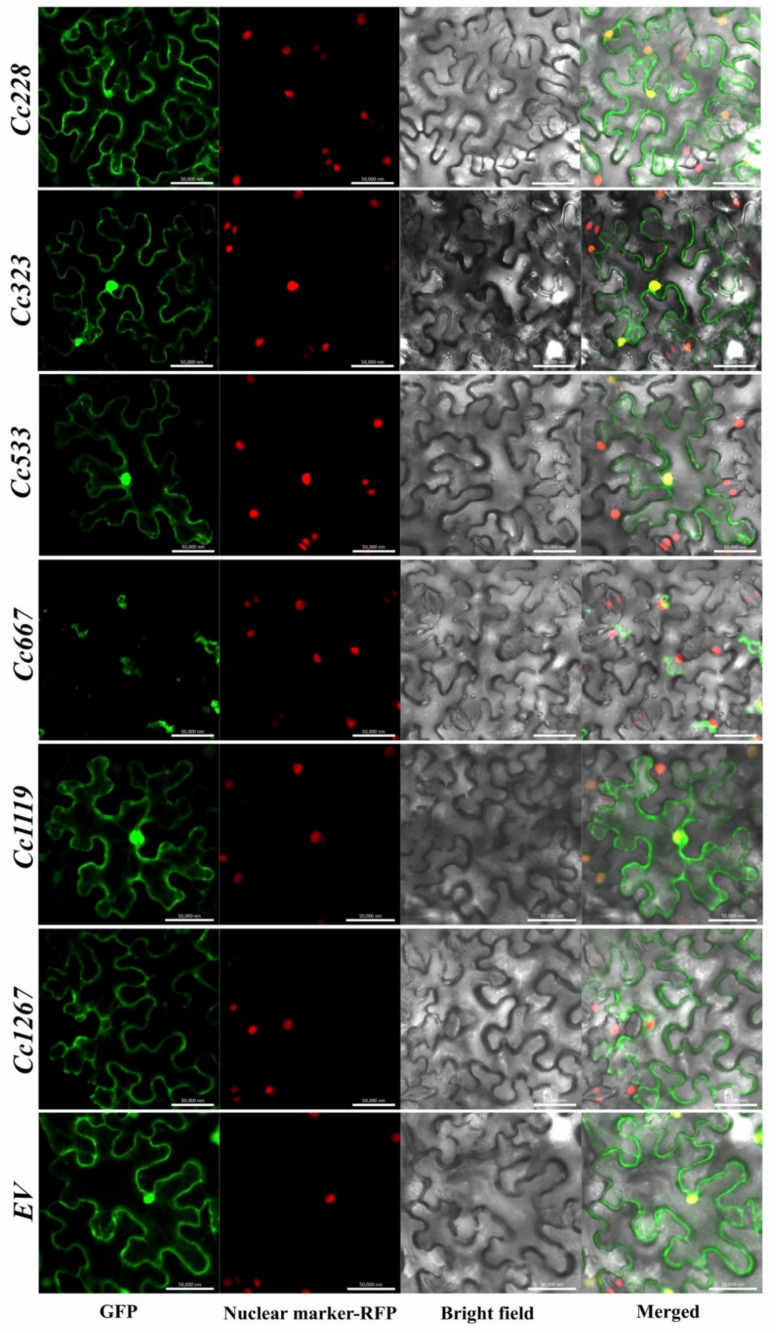
Subcellular localization of six CEPs based on confocal microscope. GFP: GFP-tagged CEPs were transiently expressed in *N. benthamiana* leaves; Nuclear marker-RFP: RFP-tagged nuclear of *N. benthamiana* epidermal cells; Bright field: this view shown the outline of the *N. benthamiana* epidermal cells; Merged: the view of samples under two excitation light. Bars = 50 μm (EV: empty vector; CEPs: candidate effectors proteins; GFP: green fluorescent protein; RFP: red fluorescent protein).

To determine the subcellular localization of *Cc228*, *Cc323*, *Cc533*, *Cc667*, *Cc1119*, and *Cc1267*, the pBin-eGFP vector was fused with these six CEPs (lacking the signal peptide) and transiently coexpressed in *N. benthamiana* epidermal cells using agroinfiltration. GFP fluorescence was visualized using confocal microscopy in *N. benthamiana* epidermal cells. There are three main patterns of GFP distribution in epidermal cells (Figure 7): nuclear, cytoplasmic, and cytomembrane. The specific results are shown in Table 1. Four of them localize to the nuclear + cytoplasmic + cytomembrane. However, *Cc667* and *Cc1267* showed different results and localized to the cytoplasmic and karyolemma + cytoplasmic + cytomembrane, respectively.

## 4. Discussion

Anthracnose is an important plant disease at both the preharvest and postharvest stages in tropical and subtropical regions, where tea plants are widely distributed [43]. *Colletotrichum* spp. has been reported as one of the most widely distributed fungal genera, and fungal conidia are spread by wind, water, and even insects [44]. Through transcriptome sequencing at different growth stages of *Colletotrichum* spp., previous studies have largely mastered the characteristics and infection strategies of phytopathogenic fungi [5,13,14,24]. Many genes relevant to the metabolic pathway function in the penetration and development of secondary hyphae have been identified, including melanization-dependent genes, cytochrome P450 enzyme genes, fatty acid genes, and cytotoxic activity genes [5,39]; however, comprehensive gene exploration in different infection stages is still necessary to discover the primary interaction between *Colletotrichum* and hosts.

Pathogen infection begins when the conidia are suspended on the plant surface. Within 8 hpi (or even longer to 12 hpi), the conidia of *C. camelliae* begin to germinate and colonize tea leaves to form specific dome-shaped dark appressoria, which is the most important structural function in the process of invasion [2,45]. Through KEGG analysis, we found an interesting phenomenon which was that the tyrosine metabolism pathway was enriched in the App_YES vs. CIH_NO and App_YES vs. IL_NO groups but absent in the App_YES vs. GT_NO group. In bacterial *Burkholderia cenocepacia*, tyrosine metabolism was suggested to affect biofilm formation, growth under nutritional deprivation, and pathogenicity [46]. However, relevant research has rarely been explored in the *Colletotrichum* genus. In addition, based on previous experience with transcriptome sequencing of *Colletotrichum* spp., many genes that can enhance infectivity and pathogenicity will be overexpressed at this phase, such as secondary metabolism, melanin biosynthesis, CAZys, cAMP, and MAPK signalling pathways [5,24,47].

In multiple pathogens, the MAPK and cAMP pathways play important roles in diverse physiological processes, such as pathogen growth, germination, cell wall integrity, and pathogenicity [48,49,50], and they are also reported play critical roles in both appressorium structure formation and melanin biosynthesis in *Colletotrichum* spp. [11,51,52]. In this study, we observed that the cAMP- and MAPK-relevant genes were upregulated at the App and CIH stages, confirming the critical function in the process of *C. camelliae* infection. We also noticed that several cAMP- and MAPK-annotated genes showed high expression at the Con stage but low expression at the GT stage and were subsequently upregulated at the App stage (Figure 5). MAPK and cAMP regulate morphogenesis and appressorium formation [50,53]. Morphological observation shows that *C. camelliae* also exhibits clear morphological changes. However, how this change is related to the downregulation of cAMP and MAPK has not been determined. In addition, we speculate that the biological process regulated by these highly expressed genes provides conidia with the ability to attach to the plant surface. This notable observation suggests that there may be special physiological processes between the Con and GT stages.

After the conidia stick into the plant leaf surface, the pathogenic fungus needs PCWDEs to degrade the plant cell walls and facilitate penetration in the next step. Moreover, this process can also help pathogens elude PTI from plants [54,55]. The CAZys contain most of the putative PCWDEs, such as the CE family, GH family GH3, GH31, and pectate lyases PL1 and PL3 [42]. However, the distribution and expression of CAZys in different *Colletotrichum* spp. are completely different. For example, in the necrotrophy stage of *C. graminicola* and *C. higginsianum*, the *C. graminicola* active more CAZys than *C. higginsianum*, including 22 GH61 oxygenases dependent on copper, which act in concert with classical cellulases to enhance lignocellulose hydrolysis [56,57]. In *C. fructicola* referred to by Liang et al. [24], the researchers predicted 1129 CAZys, and 422 of them differentially expressed. These features reflect the different infection strategies of different *Colletotrichum* spp. to degrade plant cell walls. More importantly, this finding also reflects their host preference. *C. graminicola* can specifically cause maize anthracnose, which belongs to the monocot. Conversely, *C. higginsianum* has more hosts, which are widely distributed in cruciferous crops and dicots, such as *Arabidopsis thaliana* [58]. The dicot cell wall is rich in pectin, while the monocot cell wall is rich in hemicellulose [59]. Meanwhile, the number of pectin-degrading enzymes of *C. higginsianum* is over two times that of *C. graminicola.* Tea leaves have solid physical structures, especially waxy layers [60]. In addition, the leaves are also enriched in polyphenols and caffeine, which are important for pathogen defence [61,62]. These findings indicated that the different *Colletotrichum* spp. have evolved diverse infection strategies for different plant hosts. In this study, we noticed that several GHs, GTs, and CEs showed high expression at the Con stage compared to the GT stage and then upregulated at the App stage, and the PLs only showed extremely high expression at the IL stage, which is entirely different from other *Colletotrichum* spp. This remarkable phenomenon also occurs in the cAMP and MAPK pathways, which indicates the uniqueness and specificity of *C. camelliae*.

After pathogens overcome the physical barriers of the plant surface, the effectors are secreted into the cytoplasm to increase nutrient availability for pathogens, which also triggers the plant ETI response and induces hypersensitive response and host cell death [53]. The secreted proteins are known as effectors that can boost the pathogenicity of fungal pathogens. In this study, we identified 29 non-annotated secreted proteins that showed high expression at App CIH stages as CEPs. The expression of 23 CEPs was confirmed using qRT-PCR assay in planta (Figure 6). A previous study revealed that the C-terminus is important for recognition with host transcription factors [37,63]. As a result, we used N-terminal fusion based on the rationale that tags are removed from the signal peptide. *Colletotrichum* spp. fungi require effectors to suppress the immune system of plants, and necrotrophic effectors are needed to kill plant cells. Subcellular localization is one of the most important research approaches for CEPs, which is helpful to preliminarily determine the functional position in the host. For instance, in *Magnaporthe oryzae*, the cytoplasmic effector AvrPiz-t interacts with the rice RING E3 ubiquitin ligase APIP6 and inhibits its activity to suppress an oxidative burst and PTI response [64]. In addition, many candidate effectors were revealed through genomic and transcriptome studies, which function in the whole process of infection. Our data showed that 6 CEPs are located in the nucleus, cytoplasm, cytomembrane, and karyolemma. However, further experimental screens are warranted to verify the specific functions of these CEPs.

## 5. Conclusions

This study compared the differential gene expression of five growth stages of *C. camelliae* to reveal the initial infection mechanism using transcriptomic analysis. DEGs indicated the obvious variation of melanin biosynthesis-relevant genes and PCWDEs, which are significant to pathogen penetration. The GO enriched DEGs showed that the gene and protein functions were centralized to the cellular process at the GT, App and CIH stage but the molecular function at the IL stage. The novel CEP prediction provided a new direction for research investigating virulence effectors in *C. camelliae*.

## Figures and Tables

**Table 1:** Subcellular localization assay results of six CEPs (candidate effector proteins).

Name	Subcellular Localization
*Cc228*	Nuclear + Cytoplasmic + Cytomembrane
*Cc323*	Nuclear + Cytoplasmic + Cytomembrane
*Cc533*	Nuclear + Cytoplasmic + Cytomembrane
*Cc667*	Cytoplasmic
*Cc1119*	Nuclear + Cytoplasmic + Cytomembrane
*Cc1267*	Karyolemma + Cytoplasmic + Cytomembrane

## Supplementary Materials

The following are available online at https://www.mdpi.com/2218-273X/10/5/782/s1, Figure S1: The Pearson correlation analysis of the samples, Figure S2: The number of up-regulated and down-regulated DEGs was compared with App at different growth stages, Figure S3: (**a**) KEGG analysis of up-regulated DEGs in four comparable groups; (**b**) GO analysis of up-regulated DEGs in four comparable groups, Figure S4: Venn diagram of up-DEGs in GT/App, CIH/App and IL/App, Figure S5: CAZys relevant gene expression heat map in different growth stage based on the FPKM, Table S1: Primers were used for qRT-PCR assay in vivo of CEPs, Table S2: Primers were used for cloning of six CEPs, Table S3: Primers were used for seamless cloning of pBin-eGFP vector construction, Table S4: The reagent of *agrobacterium tumefaciens* infection solution, Table S5: The quality of the transcriptomic data output, Table S6: GO enrichment analysis information of four comparison group, Table S7: The genes information of KEGG analysis based on whole DEGs between four comparison groups, Table S8: The genes information of KEGG analysis of specific up-DEGs of GT, CIH, IL compare to App, Table S9: The FPKM value of genes that relevant to melanin biosynthesis, Table S10: The FPKM value of genes that relevant to cAMP and MAPK signaling pathway, Table S11: The FPKM value of genes that relevant to CAZys, Table: S12: The FPKM value of genes that relevant to candidate effector proteins, Table S13: The sequence information of six candidate CEPs. Note: Bold italics are signal peptides sequence.
