# Supplementary material for: Lifestyle Characteristics and Gene Expression Analysis of Colletotrichum camelliae Isolated from Tea Plant [Camellia sinensis (L.) O. Kuntze] Based on Transcriptome
Source: Biomolecules. 2020 May 18;10(5):782. doi: 10.3390/biom10050782 (PMC7278179; doi:10.3390/biom10050782)
Supplement: Supplementary file 1 [file biomolecules-10-00782-s001.zip › supplementary/Fig S and Table S legend.docx]

**Fig S1.** The Pearson correlation analysis of the samples

**Fig S2.** The number of up-regulated and down-regulated DEGs was compared with App at different growth stages.

**Fig S3.** **a.** KEGG analysis of up-regulated DEGs in four comparable groups. **b.** GO analysis of up-regulated DEGs in four comparable groups.

**Fig S4.** Venn diagram of up-DEGs in GT/App, CIH/App and IL/App groups

**Fig S5.** CAZys relevant genes expression heat map in different growth stage based on the FPKM.

**Table S1:** Primers were used for qRT-PCR assay in vivo of CEPs.

**Table S2:** Primers were used for cloning of six CEPs.

**Table S3:** primers were used for seamless cloning of pBin-eGFP vector construction.

**Table S4:** the reagent of agrobacterium tumefaciens infection solution.

**Table S5:** The quality of the transcriptomic data output.

**Table S6:** GO enrichment analysis information of four comparison group.

**Table S7:** The genes information of KEGG analysis based on whole DEGs between four comparison groups.

**Table S8:** The genes information of KEGG analysis of specific up-DEGs of GT, CIH, IL compare to App.

**Table S9:** The FPKM value of genes that relevant to melanin biosynthesis.

**Table S10:** The FPKM value of genes that relevant to cAMP and MAPK signaling pathway.

**Table S11:** The FPKM value of genes that relevant to CAZys.

**Table: S12:** The FPKM value of genes that relevant to candidate effector proteins.

**Table S13:** The sequence information of six candidate CEPs. Note: Bold italics are signal peptides sequence.
