# Supplementary material for: Lifestyle Characteristics and Gene Expression Analysis of Colletotrichum camelliae Isolated from Tea Plant [Camellia sinensis (L.) O. Kuntze] Based on Transcriptome
Source: Biomolecules. 2020 May 18;10(5):782. doi: 10.3390/biom10050782 (PMC7278179; doi:10.3390/biom10050782)
Supplement: Supplementary file 1 [file biomolecules-10-00782-s001.zip › supplementary/Tables/Table S1_S2_S3_S4_S5.docx]

Table S1

| qPCR | 5’ 3’ |
| --- | --- |
| Ccnew1F (internal control) | CGTGGCTTTGAAATCAGACC |
| Ccnew1F (internal control) | CCATCTCGTGACTAGGAGCAA |
| Cc228F | GGTGGTGGTGAAGATGATAC |
| Cc228R | CAGCAGTCCAGCAATCAA |
| Cc2489F | GTAGGGTTAGGACTACCAAGA |
| Cc2489R | ACGTTGCCCTCAGAAATG |
| Cc323F | TCTCCTCAACGGTCTTCTC |
| Cc323R | TTACTCCTCGTCATCAGCA |
| Cc11F | CAGTCAAGAGATGCTCCAAC |
| Cc11R | GGACACGTACTTCCTGTAATC |
| Cc1267F | CGGTATCGTGTCTAACCAATC |
| Cc1267R | CGTCTCTGTCGATCTCATAATC |
| Cc164F | CATCAAGTGGGATGATGGAG |
| Cc164R | AGTGGAGGGTGTCGTTTA |
| Cc582F | GCGATGCGGTACTCTCT |
| Cc582R | AGAGCCCAAAGTCCATCA |
| Cc1173F | TACCTTGGTGTCGTGGATAA |
| Cc1173R | CGTCCAAGTAGATCGTGAAAC |
| Cc1437F | CGAGCACATCCAGACATC |
| Cc1437R | CCCACAAACAGACTCATCATA |
| Cc1158F | GAGGGCTGTCTTTGACTATTT |
| Cc1158R | TTCTCAACCCTCCCATCA |
| Cc2106F | CCGTCGAGTTCCACTTCTA |
| Cc2106R | GCGCAGTAGAACCACTTG |
| Cc224F | ATACCAGGAGCACGGATTA |
| Cc224R | GTACTGAAGCCCAAACAGTAG |
| Cc967F | CCCAAGATACTTCTCACCTTATT |
| Cc967R | GTCAACCTCTCTCAACTCATAC |
| Cc970F | ACAAGAAGTCGTCTTCGATAAG |
| Cc970R | AGTGAGGGTCGAAGTTATAGT |
| Cc52F | GCTACATCATGGTTCACCTC |
| Cc52R | GTCTTTCCAGACTCGTCAAG |
| Cc241F | CTCTGCCAGTACAGTCTATGA |
| Cc241R | GGATGAAATCGGCTCGTAAG |
| Cc755F | GGTGCCGAGATTTGGATTT |
| Cc755R | TGCGTGTTGATGGTGAAG |
| Cc1928F | CAGGTCCTCAACCACAAC |
| Cc1928R | GACGCTGACATCCTTGAC |
| Cc2589F | CTTCGTCGAGCTCAAGTC |
| Cc2589R | CCGTCAAAGTCGATCTCATAG |
| Cc533F | GCTTCTGTTGACGATGCT |
| Cc533R | GTTAGCCTTCTTCGTCTTCTT |
| Cc1077F | TCTCCTACTGATCCCAACTAC |
| Cc1077R | GGAGACGCTTCATTCTCATC |
| Cc109F | GGCATGGATTTCACCTTCT |
| Cc109R | CGGCATCATCCTGCATAAA |
| Cc1119F | AAGCGCTCCATCACCTA |
| Cc1119R | AGAGGTGGACTTGAACCA |
| Cc166F | TACTCTGCCAGTCGTCTATC |
| Cc166R | CAAGAACACCACCAGCTATT |
| Cc503F | CAACCGCAACTGGAACTA |
| Cc503R | GAGTGGAAGTCGATGAAGAG |
| Cc667F | AGACCGCTGAGATCTACTC |
| Cc667R | CGTTGTAGTTGGGCTTCTC |
| Cc1927F | ACGTCTGGGTCGTATAGTC |
| Cc1927R | GCACGCAGAACAGGTAAT |
| Cc2235F | CGACAACGGCAAGATCTAC |
| Cc2235R | CTCGACGATCATGAGGTACT |
| Cc324F | GAAGATGTCAGCCTCTCTTTC |
| Cc324R | GATGCCGGGACCATAGA |

Table S2

| Cc228 | F: 5’ATGCTCCCCAAACTCCTCCT 3’; R: 5’CAGATAACCAGGGGCACAGC3’ |
| --- | --- |
| Cc323 | F: 5’ ATGAAGAGCTCTTTCATCCTCTC 3’; R: 5’ TTACTCCTCGTCATCAGCAGC 3’ |
| Cc533 | F: 5’ ATGCATTACCCTTCTTTCGG 3’; R: 5’ TTAGGCGGTGAACGCAAC 3’ |
| Cc667 | F: 5’ ATGGAGCCCAAGAACACTTTTG 3’; R: 5’ ATGGAGCCCAAGAACACTTTTG 3’ |
| Cc1119 | F: 5’ ATGGTTGCCAAAGCCCTC 3’; R: 5’ TTAACCGACGGTGCAGC 3’ |
| Cc1267 | F: 5’ ATGAAAATGAGATTCACGGACGC 3’; R: 5’ TCATAGCTCATCCTTGACTTCAGG 3’ |

Table S3

| GeneID | Forward primer（5’ - 3’） |  |
| --- | --- | --- |
| 228_eGFP - F  228_eGFP - R  323_eGFP - F  323_eGFP - R  533_eGFP - F  533_eGFP - R  667_eGFP - F  667_eGFP - R  1119_eGFP - F  1119_eGFP - R  1267_eGFP - F  1267_eGFP - F | TACAAGGGTACCCCCatgGACTCCTCCTCCCCCTA  CATCTAGAGGATCCGTCAGATAACCAGGGGCACAG  TGTACAAGGGTACCCatgCTCCCCACCCAGACCGA  CATCTAGAGGATCCGTTACTCCTCGTCATCAGCAGCGT  TGTACAAGGGTACCCatgTTCAACCTCATCCCCAATG  CATCTAGAGGATCCGTTAGGCGGTGAACGCAACGG  TACAAGGGTACCCCCatgCACACCTGGGTTGAGCAGC  CATCTAGAGGATCCGGATGTGAGGGTTGAAGTTCC  TGTACAAGGGTACCCatgACCTGCCCCCTCTCGGT  CATCTAGAGGATCCGTTAACCGACGGTGCAGCCACC  TACAAGGGTACCCCCatgCTCAATGTTGGCCCAGA  CATCTAGAGGATCCGTCATAGCTCATCCTTGACTTCA |  |

Table S4

| Reagent | Usage amount |
| --- | --- |
| MES stock（500 mM） | 1 mL |
| Acetosyringone（1 M）  MgCl_2_·6H_2_O（500 mM） | 7.5 μL  1 mL  ddH_2_O up to 50 mL |

Table S5

| Sample name | Raw reads | Clean reads | Clean bases | Error rate (%) | Q20(%) | Q30(%) | GC content |
| --- | --- | --- | --- | --- | --- | --- | --- |
| Con1 | 68272580 | 65924456 | 9.89G | 0.03 | 96.57 | 91.56 | 57.09 |
| Con2 | 68972496 | 67489602 | 10.12G | 0.03 | 96.44 | 91.31 | 57.21 |
| Con3 | 67842310 | 66443124 | 9.97G | 0.03 | 96.42 | 91.28 | 57.10 |
| GT1 | 65384120 | 64038786 | 9.61G | 0.03 | 96.50 | 91.39 | 58.13 |
| GT2 | 57854796 | 56143566 | 8.42G | 0.03 | 96.41 | 91.22 | 58.07 |
| GT3 | 61872178 | 60096884 | 9.01G | 0.03 | 96.26 | 90.92 | 58.05 |
| IL1 | 73493834 | 71776712 | 10.77G | 0.03 | 95.62 | 89.33 | 50.16 |
| IL2 | 76311072 | 73088370 | 10.96G | 0.03 | 95.64 | 89.47 | 52.86 |
| IL3 | 73756304 | 71927190 | 10.79G | 0.03 | 95.62 | 89.36 | 49.35 |
| App1 | 53660940 | 52285718 | 7.84G | 0.03 | 96.52 | 91.17 | 57.13 |
| App2 | 46790820 | 45674400 | 6.85G | 0.03 | 95.92 | 90.06 | 56.87 |
| App3 | 46055660 | 44327646 | 6.65G | 0.03 | 96.20 | 90.55 | 56.76 |
| CIH1 | 67989352 | 66565704 | 9.98G | 0.03 | 96.70 | 91.65 | 56.85 |
| CIH2 | 71783064 | 70149382 | 10.52G | 0.03 | 96.65 | 91.59 | 56.71 |
| CIH3 | 66279794 | 64968962 | 9.75G | 0.03 | 96.94 | 92.20 | 56.81 |
