# Supplementary figures and images for: Lifestyle Characteristics and Gene Expression Analysis of Colletotrichum camelliae Isolated from Tea Plant [Camellia sinensis (L.) O. Kuntze] Based on Transcriptome
Source: Biomolecules. 2020 May 18;10(5):782. doi: 10.3390/biom10050782 (PMC7278179; doi:10.3390/biom10050782)

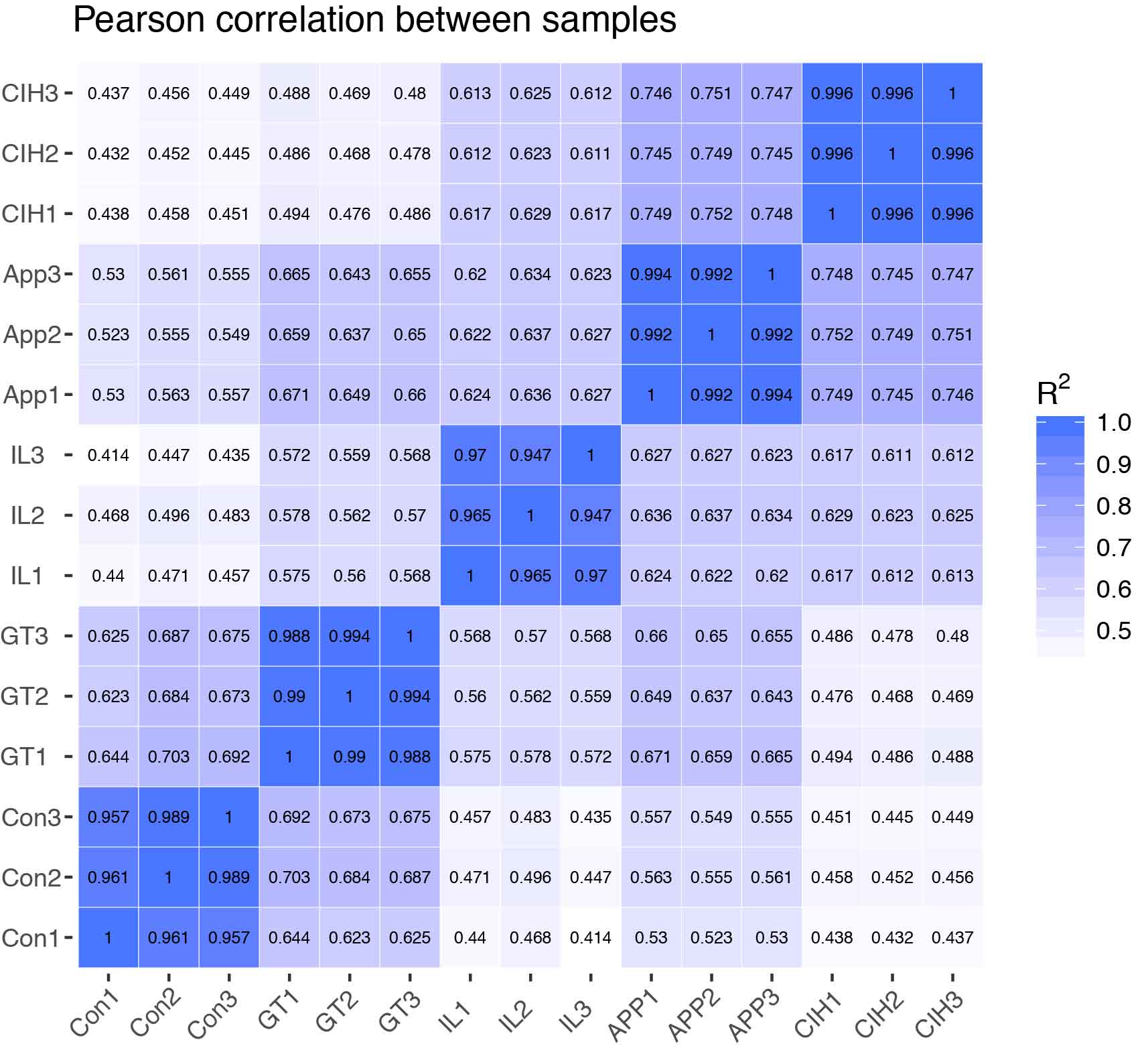

Supplement: Supplementary file 1 [file biomolecules-10-00782-s001.zip › supplementary/Figures/Fig S1.jpg]

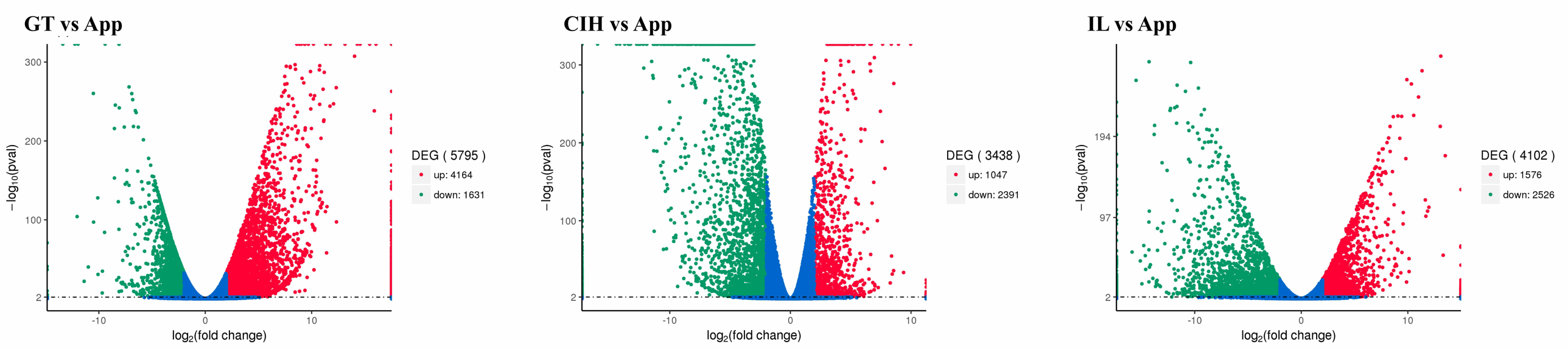

Supplement: Supplementary file 1 [file biomolecules-10-00782-s001.zip › supplementary/Figures/FIG S2.jpg]

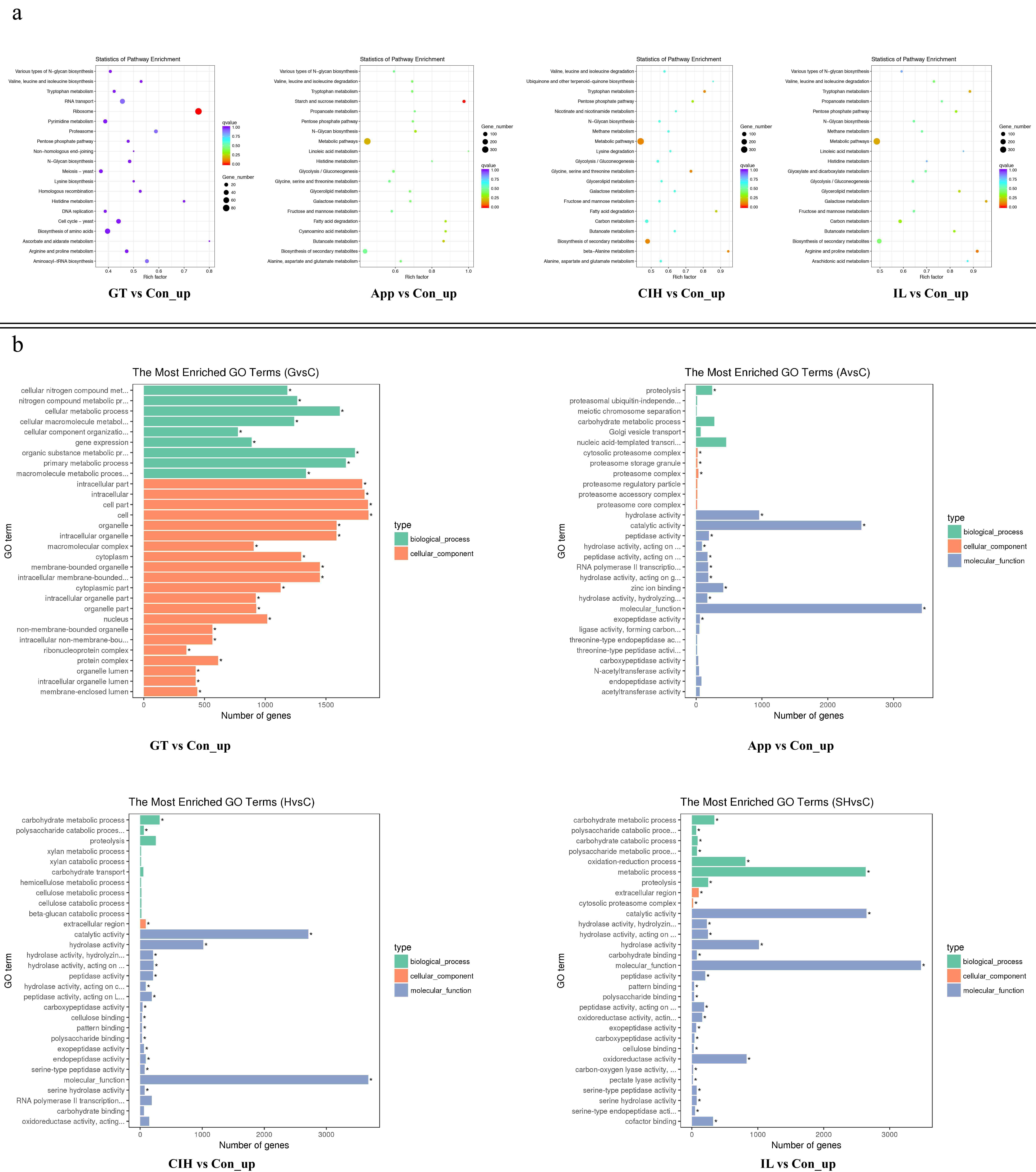

Supplement: Supplementary file 1 [file biomolecules-10-00782-s001.zip › supplementary/Figures/FIG S3.jpg]

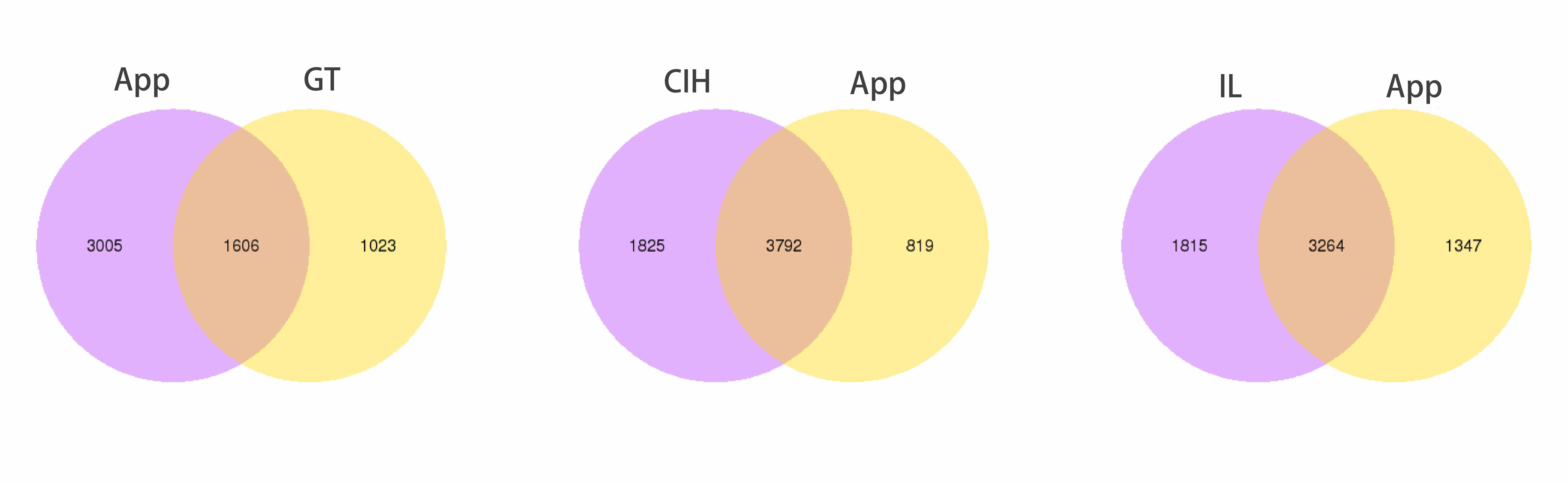

Supplement: Supplementary file 1 [file biomolecules-10-00782-s001.zip › supplementary/Figures/Fig S4.jpg]

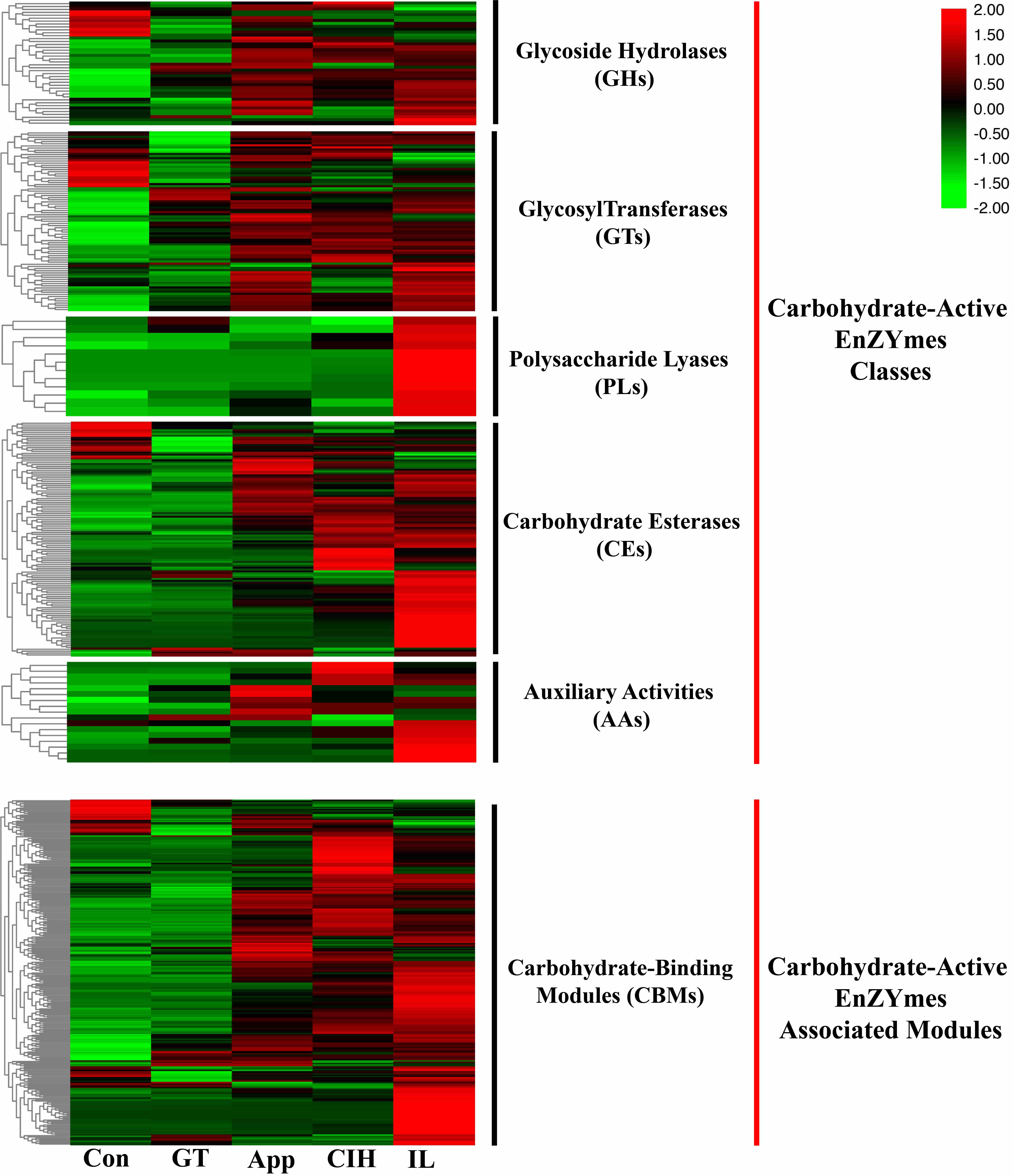

Supplement: Supplementary file 1 [file biomolecules-10-00782-s001.zip › supplementary/Figures/FIG S5.jpg]
